# Supplementary material for: Intravitreal Administration of Retinal Organoids-Derived Exosomes Alleviates Photoreceptor Degeneration in Royal College of Surgeons Rats by Targeting the Mitogen-Activated Protein Kinase Pathway
Source: Int J Mol Sci. 2023 Jul 27;24(15):12068. doi: 10.3390/ijms241512068 (PMC10419150; doi:10.3390/ijms241512068)
Supplement: Supplementary file 1 [file ijms-24-12068-s001.zip › ijms-2490025 - supplementary.pdf]

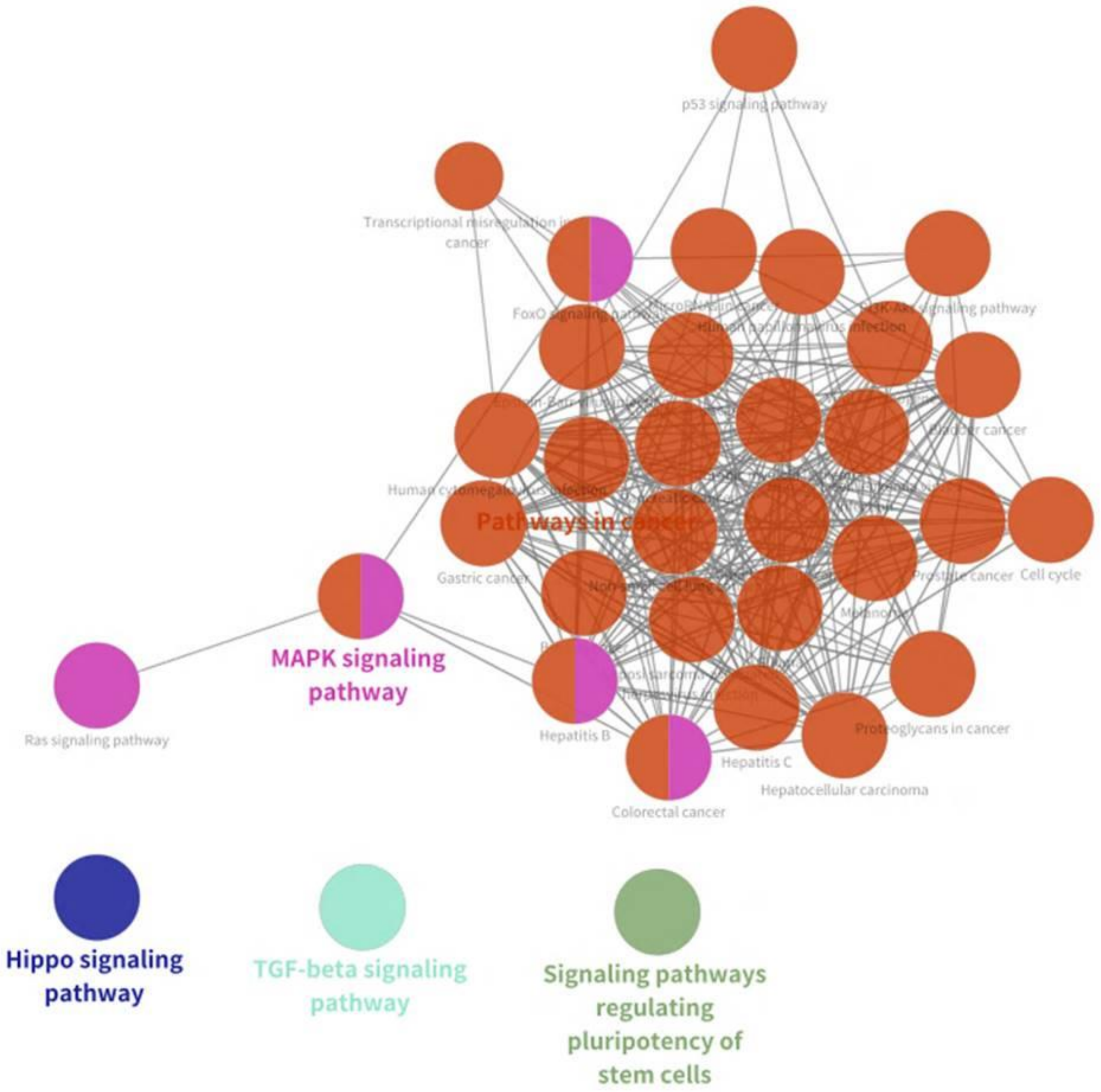

Figure S1. Clustering and network analysis of pathways which were enriched the 293 shared target genes of 10 or more miRNAs in Exo-ROs. The analysis was performed using ClueGO package (version 2.5.9) in Cytoscape software (version 3.5). The key pathway of the target genes of Exo-RO miRNAs is the MAPK signaling pathway.

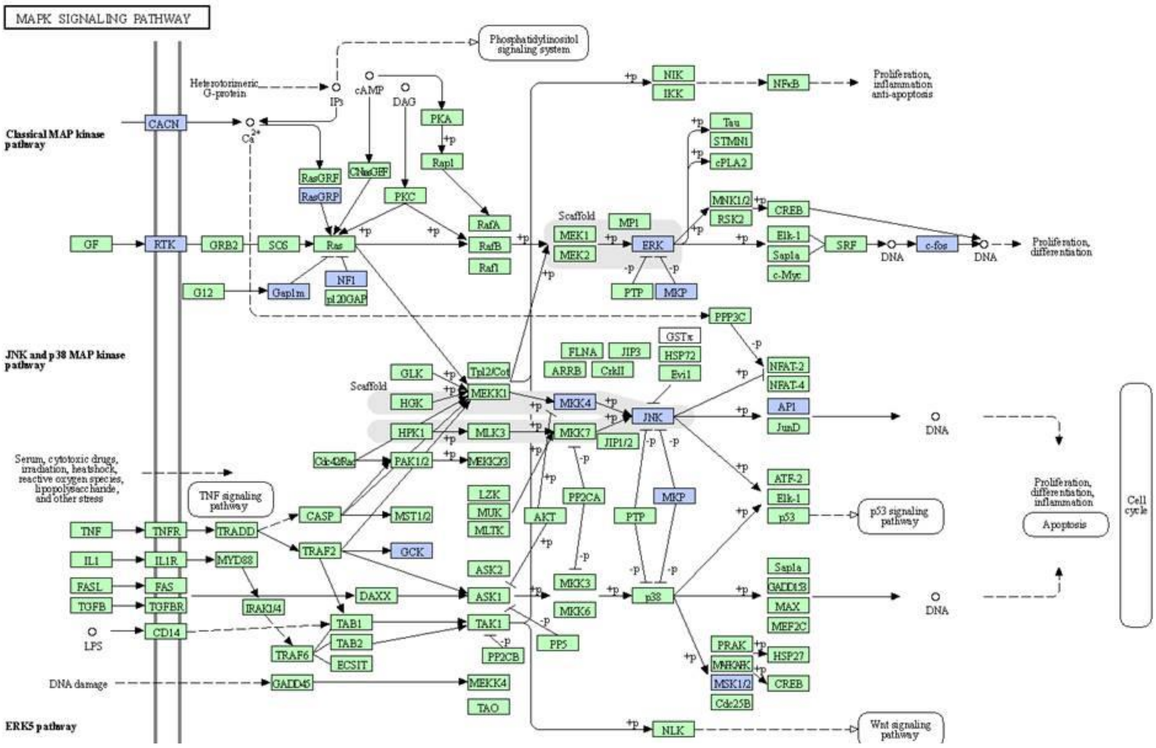

Figure S2. The Kyoto Encyclopedia of Genes and Genomes pathway for mitogen-activated protein kinase signaling pathway. Genes within the significant differential expression in exosomes from retinal organoids treated group are shown in blue.

miRNA	Abundance rank in Exo-RO	DUSP16	EGFR	FOS	MAP2K4	MAPK1	MAPK8	MAPT	NF1	RASA2	RASGRP1	RPS6KA5	TEK
hsa-mir-122-5p	1		■*						■				
hsa-mir-148a-3p	4	■		■		■					■		
hsa-let-7a-5p	5			■	■	■	■	■				■	■
hsa-let-7b-5p	6	■	■	■	■	■*	■	■		■	■	■	■
hsa-let-7c-5p	7	■			■	■	■	■				■	
hsa-let-7f-5p	8	■			■	■	■					■	
hsa-mir-125a-5p	9									■			
hsa-mir-26a-5p	12			■			■				■		
hsa-mir-128-3p	13	■		■	■	■			■			■*	
hsa-mir-27b-3p	14	■			■	■	■		■*			■	
hsa-mir-126-3p	15			■		■			■				
hsa-mir-10a-5p	16									■			
hsa-let-7e-5p	18	■			■	■	■		■*			■	
hsa-mir-21-5p	19	■	■*		■	■			■		■*		■
hsa-mir-30d-5p	20	■					■*			■			

■ Tarbase

* Mirtarbase (with publications)

Figure S3. MicroRNAs (miRNAs) targeting 12 genes contained in the Gene Ontology term "mitogen-activated protein kinase pathway" among 20 miRNAs abundant in exosomes from retinal organoids.
